# Risk of Complications After Breast Surgery in Radiated Patients Using the Closed Incision Negative Pressure Therapy Dressings Compared to Standard-of-Care Dressings

**DOI:** 10.1093/asjof/ojad027.013

**Published:** 2023-04-14

**Authors:** Khaled Alameddine, Omar Mohamed, Austin Chen, Christin Harless

**Affiliations:** Mayo Clinic, Rochester, MN; Mayo Clinic, Rochester, MN; Mayo Clinic, Rochester, MN; Mayo Clinic, Rochester, MN

## Abstract

**Goals/Purpose:**

Patients with adjuvant radiotherapy are significantly more likely to have complications such as infections and wound dehiscence following breast surgery.^1^ Closed incision negative pressure wound therapy (cINPT) holds incision edges together, prevents external contamination and removes exudate, promoting tensile strength and increased collagen recruitment at the incision site compared to the standard of care dressings.^2^ cINPT is helpful in surgical closures on high-risk patients.^3^ We evaluated the complication rates following the use of cINPT in implant-based breast reconstruction.

**Methods/Technique:**

A cohort was collected retrospectively from a single institution, from January 1, 2018 to October 31 2022. The complication rates were then compared between cINPT dressing and standard-of-care dressings on radiated patients following breast surgery.

**Results/Complications:**

The data collected consist of a total of 83 radiated breasts that underwent implant based reconstruction. cINPT was used in 41 breasts and standard-of-care dressings were used in 42 breasts.

Of the cINPT cohort, 2 patients developed seromas that required bedside aspirations (4.8%) and one patient developed an infection (2.4%) with none of the patients requiring re operations. Of the standard of care dressings cohort, 9 patients developed seromas (21.4%), and 3 patients developed infections (7.1%), of which 3 patients required reoperations (7.1%).

**Conclusion:**

Patients with prior radiation who received cINPT had lower risk of developing complications compared to patients with prior radiation who received standard-of-care dressings.

